# Surface Nanostructure Effects on Dopamine Adsorption and Electrochemistry on Glassy Carbon Electrodes

**DOI:** 10.1021/acs.jpcc.2c02801

**Published:** 2022-07-29

**Authors:** Dalia L. Swinya, Daniel Martín-Yerga, Marc Walker, Patrick R. Unwin

**Affiliations:** † Department of Chemistry, University of Warwick, Coventry CV4 7AL, United Kingdom; ‡ Department of Physics, University of Warwick, Coventry CV4 7AL, United Kingdom

## Abstract

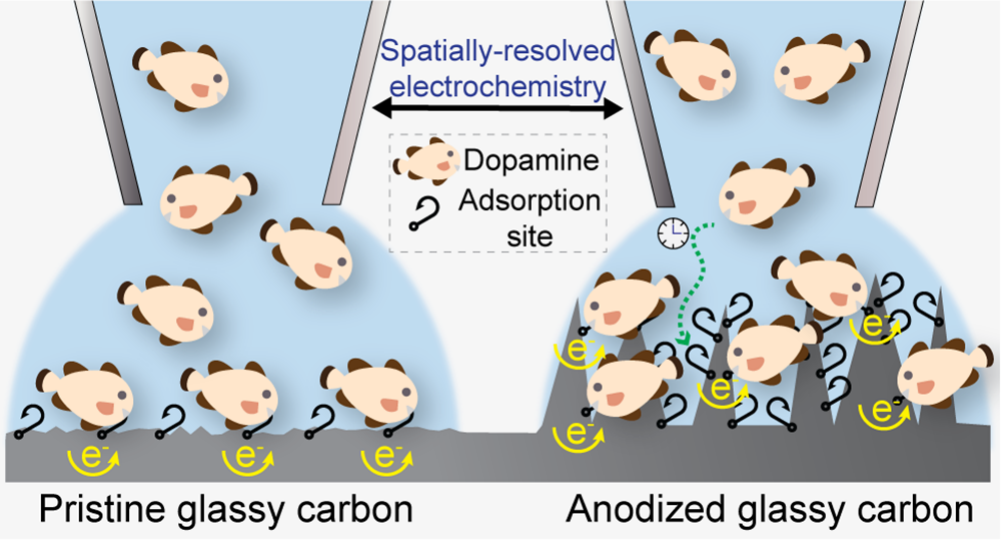

Dopamine (DA) adsorption and electron-transfer kinetics are strongly sensitive to the structure and composition of carbon electrodes. Activation of carbon surfaces is a popular method to improve DA detection, but the role of carbon structural features on DA behavior remains uncertain. Herein, we use scanning electrochemical cell microscopy (SECCM) for local anodization of glassy carbon (GC) electrodes in acid media followed by electrochemical imaging of DA adsorption and electrochemistry covering both unmodified and anodized GC regions of the same electrode. Electrochemical measurements of adsorbed DA involve the delivery of DA from the SECCM meniscus (30 μM) for 1 s periods followed by voltammetric analysis at a reasonable sweep rate (47 V s^–1^). This general approach reduces effects from interelectrode variability and allows for considerable numbers of measurements and statistical analysis of electrochemical data sets. Localized electrode activity is correlated to surface structure and chemistry by a range of characterization techniques. Anodization enhances DA electron-transfer kinetics and provides more sites for adsorption (higher specific surface area). A consequence is that adsorption takes longer to approach completion on the anodized surface. In fact, normalizing DA surface coverage by the electrochemical surface area (ECSA) reveals that adsorption is less extensive on anodized surfaces compared to as-prepared GC on the same time scale. Thus, ECSA, which has often been overlooked when calculating DA surface coverage on carbon electrodes, even where different activation methods would be expected to result in different surface roughness and nanostructure, is an important consideration. Lower graphitic and higher oxygen content on anodized GC also suggest that oxygen-containing functional groups do not necessarily enhance DA adsorption and may have the opposite effect. This work further demonstrates SECCM as a powerful technique for revealing surface structure–function relationships and correlations at heterogeneous electrodes.

## Introduction

Monitoring dopamine (DA) concentration fluctuations and transmission is important in the study of several neurological and cognitive processes,^1−3^ with electrochemical detection enabling the accurate monitoring of DA levels in different biological fluids and tissues.^4−6^ DA electrochemistry involves proton-coupled electron transfer, with a two-electron two-proton oxidation, through the catechol group^7^ to form dopaminequinone (DAQ) (Figure S1).^8,9^ DAQ can then suffer a subsequent cascade of chemical/electrochemical side reactions, ultimately leading to the formation of melanin-like polymeric compounds^10^ that can result in the fouling of electrode surfaces.^11,12^

Carbon-based electrodes are very attractive for DA detection.^13^ For instance, carbon-fiber microelectrodes (CFMEs) are standard tools to carry out in vivo measurements of neurotransmitters^6^ with high spatiotemporal resolution through fast-scan cyclic voltammetry.^14^ Other carbon-based materials such as glassy carbon (GC),^15^ carbon nanotubes,^16,17^ screen-printed graphite,^18^ boron doped diamond,^19^ and graphene^20^ have also been reported as electrodes for DA analytical determination. DA adsorption, electrochemical kinetics, and fouling are strongly affected by the nature of the carbon electrode surface.^11,12^ In this regard, activation procedures, which are known to change surface properties and composition, are typically used to improve DA detection.^21,22^ Such activation can affect the electron-transfer kinetics and adsorption,^23−25^ thereby decreasing overpotentials and improving sensitivity and resolution. This ability to tailor the carbon surface structure has been widely exploited to study DA response on GC electrodes activated by different methods such as heat,^26^ laser,^27,28^ and plasma^29^ treatments. Surface polishing can also introduce compositional changes by increasing the content of surface oxide groups.^30^ In turn, this was believed to promote DA adsorption through electrostatic interaction and/or ion-exchange between the negatively charged surface oxide layer and the protonated amine group in DA.^31^ However, quantum chemistry calculations indicate that adsorption occurs through a noncovalent interaction between the π-system of the aryl ring and graphitized regions of the surface.^32^

Electrochemical anodization has been widely employed to activate carbon surfaces^15,31−35^ and remove surface impurities.^34^ This process generally leads to changes in the surface micro/nanostructure, as surface roughening is typically observed,^15^ due to etching of the carbon material by oxidation to CO_2_,^35,36^ resulting in increased electrode/electrolyte capacitance^37,38^ and electrochemically active surface area.^36^ Oxygen functionalities are created on the carbon surface by partial oxidation.^39^ All of these factors can influence DA adsorption and reactivity, but their individual effects are difficult to resolve, and sometimes, contradictory phenomena have been reported. For instance, GC anodization has been reported to promote catechol adsorption,^40^ although an absence of DA adsorption with a significant increase of electron-transfer kinetics has also been recently described.^32^ Specific anodization conditions, such as the applied potential, time, and electrolyte media, can also affect the GC surface structure and composition.^41^ New approaches are thus required to reveal structure–activity relationships for carbon electrodes that provide original insights into DA adsorption and electrochemistry, and enable the rational design of improved electrode surfaces for high-sensitivity detection of neurotransmitters.

Scanning electrochemical probe microscopy techniques have been used successfully to study the local activity of carbon electrodes for the electrochemical oxidation of DA^42−45^ and other neurotransmitters.^46,47^ Scanning electrochemical cell microscopy (SECCM) revealed that the basal plane of graphite showed high DA electro-oxidation activity,^42^ and measurements on HOPG with a wide range of step edge density showed that the voltammetric response (and blocking in repetitive voltammetric cycles) could be explained entirely by the electrochemistry of the basal plane alone.^11^ That graphite particles with a high degree of crystallinity promote fast electro-oxidation of adsorbed DA was further confirmed with coupled SECCM–Raman microscopy imaging of screen-printed carbon electrodes.^43^

In this work, SECCM is used to carry out local anodization of GC electrodes with a large tip (30 μm diameter) and then to produce spatially resolved electrochemical maps and movies of DA adsorption and electrochemical reactivity with a small tip (800 nm diameter), with the scan area covering both unmodified and locally anodized regions of the GC surface. This approach provides direct visualization of any changes in DA behavior, *in the same experiment*, that are readily correlated to the carbon surface structure by a range of surface characterization techniques (atomic force microscopy (AFM), Raman, energy-dispersive X-ray spectroscopy (EDS), and X-ray photoelectron spectroscopy (XPS)). SECCM is applied under conditions where the DA electro-oxidation signature is due to adsorbed DA: fast scan rates (47 V s^–1^) and low concentrations (micromolar level), where fouling should be relatively insignificant. Anodization of GC clearly enhances DA electron-transfer kinetics, although an apparent increase in DA adsorption is attributed to the effect of the increased surface area due to surface roughening. This change in surface nanostructure leads to DA adsorption taking longer to approach completion on the rougher surfaces. The correlative multi-microscopy approach outlined herein is generally applicable for the determination of structure–adsorption–activity relationships for heterogeneous electrode surfaces.

## Methods

### Materials and Chemicals

Dopamine hydrochloride (≥98%), perchloric acid (HClO_4_, 70% w/w), and phosphate-buffered saline (PBS) (0.05 M with 0.138 M NaCl, pH 7.4) were purchased from Sigma-Aldrich. Sulfuric acid (≥98%) was purchased from Fisher Scientific. Stock solutions of DA were prepared in 0.1 M HClO_4_ and stored in a fridge at 4 °C. DA solutions in PBS were prepared daily from the stock solution, and the pH was measured using a pH meter (UltraBASIC pH meter, Denver Instruments). Ultrapure water from a Millipore Milli-Q system (resistivity: 18.2 MΩ cm at 25 °C) was used throughout. High-quality glassy carbon (GC) (25 × 25 × 3 mm) was purchased from Alfa Aesar. The GC was polished before each experiment using a polishing pad with alumina slurry (particle size: 0.05 μm, Buehler). After polishing, the GC was sonicated in ultrapure water for 20 min.

### Local Electrochemical Measurements

**Figure 1:**
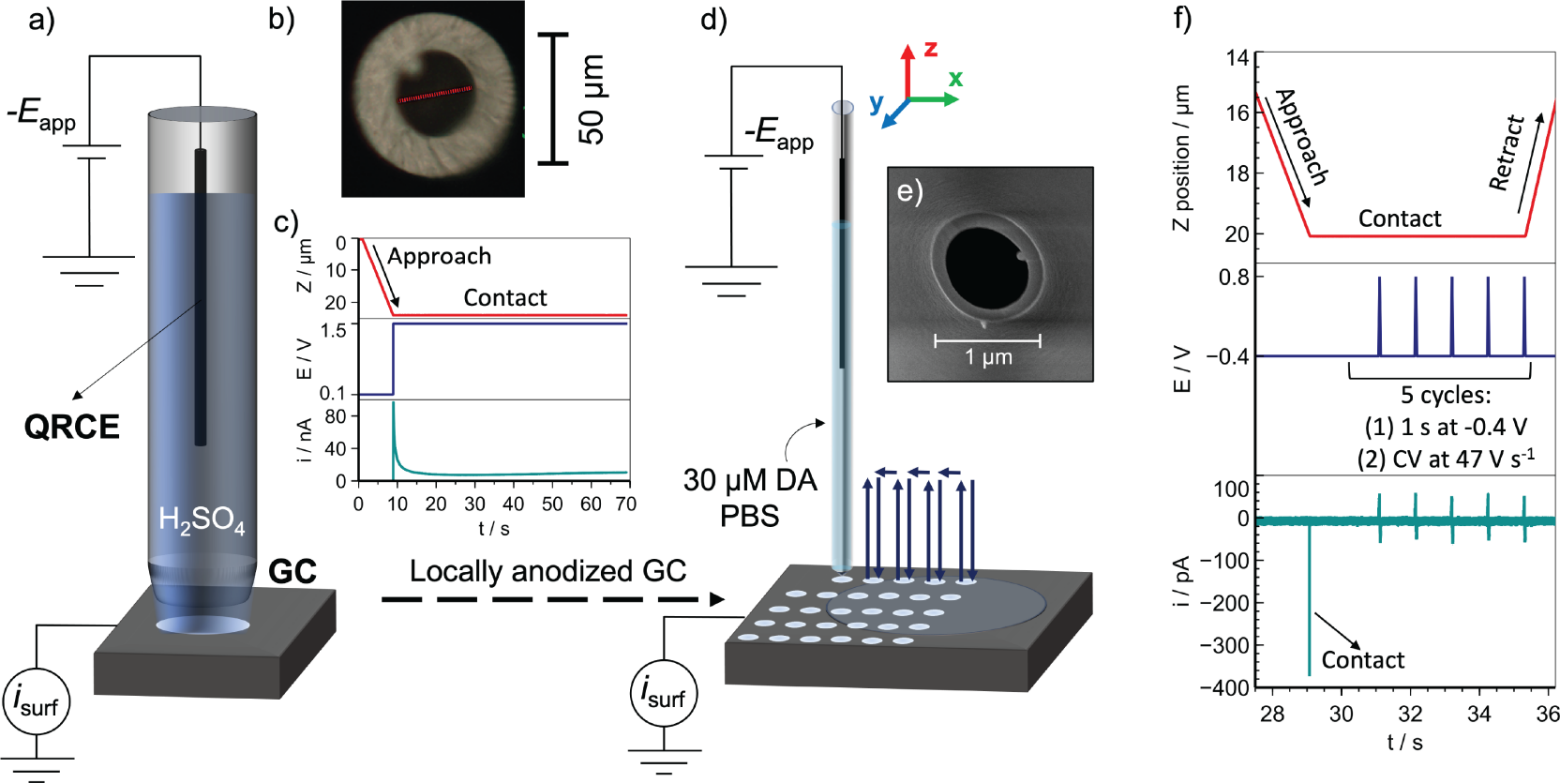
(a) Schematic of the SECCM device operated in the static mode to carry out the local anodization of the GC electrode. A single-channel pipet filled with 5 mM H_2_SO_4_ was approached to the GC surface, and a chronoamperometric experiment was applied through a constant potential (−*E*_app_) of +1.5 V vs Ag/AgCl QRCE for 60 or 300 s, measuring the surface current (*i*_surf_) flowing at the GC electrode. (b) Optical image of the end of a pipet with ca. 30 μm diameter. (c) Plots showing the variation of the pipet *z* position, the applied potential, and the measured current as a function of time during the anodization. (d) Schematic of the SECCM hopping protocol to study DA adsorption and electrochemistry on the locally anodized GC surface. (e) A pipet probe with ca. 800 nm diameter was translated to a series of predefined locations on the GC electrode to cover both pristine (polished) and anodized regions within the same experiment. (f) Plots showing the SECCM protocol in one location including the variation of the pipet *z* position to achieve meniscus–surface contact, the voltammetric program (equilibration for 1 s at −0.4 V followed by a five-times repeated sequence of 1 s at −0.4 V and then a voltammetric sweep analysis between −0.4 and + 0.8 V at 47 V s^–1^, taking ca. 50 ms; DA adsorption occurs during the periods at −0.4 V), and the typical current response as a function of time obtained.

All SECCM experiments were performed using a home-built scanning electrochemical probe microscopy workstation.^48−50^ Local anodization of the GC electrode was carried out with SECCM in the static mode at one location of the GC surface as illustrated in Figure 1a. A pipet probe with a diameter of ca. 30 μm (Figure 1b) was pulled from a single channel borosilicate capillary (BF100-50-10, Harvard Apparatus) using a PC-10 Narishige puller. An AgCl-coated Ag wire was inserted into the pipet to act as a quasi-reference counter electrode (QRCE). The pipet, filled with 5 mM H_2_SO_4_, was approached to the GC surface at 3 μm s^–1^ using a *z* piezoelectric stage (P-753.3CD, Physik Instrumente), and landing was detected by a change in the surface current (*i*_surf_) with a threshold of 11 pA (at +0.1 V vs Ag/AgCl QRCE), which indicated the formation of a liquid meniscus between the pipet and the GC surface (without contact from the pipet). After landing, GC anodization was initiated by applying a constant potential of +1.5 V vs Ag/AgCl QRCE for either 60 or 300 s (Figure 1c). The anodization potential was selected based on previously reported values.^32,34,51,52^

Spatially resolved voltammetric SECCM measurements were performed using the hopping mode (Figure 1d). A pipet probe of ca. 800 nm diameter (Figure 1e) was pulled from a borosilicate capillary (BF 120-69-10, Harvard Apparatus) using a CO_2_ laser puller (P-2000, Sutter Instruments). The pipet was filled with 30 μM DA in PBS (pH 7.4), and the SECCM procedure involved a series of predefined steps (Figure 1f) at different locations (pixels in maps) of the GC sample. Briefly, the pipet probe was approached to the GC surface at 3 μm s^–1^ until reaching an *i*_surf_ threshold of 4 pA (at −0.4 V vs AgAgCl QRCE), which indicated contact of the liquid meniscus of the pipet with the GC surface. The resulting electrochemical cell was maintained at this potential for 1 s to allow for DA adsorption followed by a cyclic voltammetry (CV) measurement at a scan rate of 47 V s^–1^ between −0.4 V (start and end) and +0.8 V (reverse potential) vs Ag/AgCl QRCE. This voltammetric process was repeated a further four times (five cycles in total), with a 1 s pause between each (at −0.4 V), so as to follow the evolution of DA adsorption. The pipet probe was then retracted and moved to a new location (pixel) at 5 μm s^–1^ to repeat the same procedure at a predefined grid of pixels separated by 5 μm. The sample was translated in the *xy* directions using a *XY* piezoelectric stage (P-621.2CD). Data acquisition was achieved using an FPGA card (PCIe-7852R, National Instruments) with a LabVIEW 2019 interface running the Warwick Electrochemical Scanning Probe Microscopy (WEC-SPM) software. A data acquisition rate of 130 μs was used, as *i*_surf_ was measured every 2 μs and averaged 64 times, with one extra iteration used to transfer data to the computer. The whole SECCM setup was placed on a passive mechanical vibration isolation platform within a Faraday cage equipped with heat sinks and acoustic foam to minimize mechanical vibration, electrical noise, and thermal drift. All experiments were performed at room temperature. Data processing and analysis were done with a Python code using SciPy libraries.^53^ Statistical analysis was conducted with Minitab 19.1 (Minitab Ltd.), with *p* values significant at the 95% confidence level (*p* < 0.05).

### Surface Characterization

An Olympus BH2 optical microscope under top-side illumination (reflection mode) equipped with a camera (PL-B782U, 4× lens, Pixelink) was used to measure the diameter of the ca. 30 μm pipet probes.

AFM was carried out using an Innova microscope (Bruker) in tapping mode with Antimony (n) doped Si probes (RFESP-75, Bruker). Scans were recorded with 512 points per line at 0.1 Hz over 10 × 10 μm^2^ of the GC electrode area. AFM images were analyzed with the Gwyddion software (v2.55, Czech Metrology Institute).

Scanning electron microscopy (SEM) was employed to measure the diameter of the ca. 800 nm pipet probes and to record images of the droplet footprints left by the liquid meniscus during the SECCM experiments. SEM images were obtained with a field emission scanning electron microscope (FE-SEM, ZEISS Gemini, Germany) at an acceleration voltage of 5 kV using the In Lens detector. Elemental quantification was carried out by EDS using the integrated detector of the SEM instrument.

Raman imaging was implemented using a Renishaw InVia Microscope with a 532 nm excitation laser at 100% nominal power (35 mW), spread out over 1000 spots, and 1800 mm^–1^ gratings. Acquisition time was 1 s by spectrum.

XPS data were collected at the Photoemission RTP (University of Warwick). GC samples were attached to an electrically conductive carbon tape and mounted onto a sample bar before being loaded into a Kratos Axis Ultra DLD spectrometer that possesses a base pressure below 10^–10^ mbar. XPS measurements were performed in the main analysis chamber, with the sample being illuminated using a monochromatic Al Kα X-ray source (hν = 1486.7 eV). Measurements were conducted at room temperature and at a take-off angle of 90° with respect to the surface parallel. Core-level spectra were recorded using a pass energy of 20 eV (resolution approx. 0.4 eV) from an analysis area of 300 × 700 μm. To exclusively cover an anodized area during XPS measurement, a region of the GC sample (diameter = ca. 5 mm) was anodized under the same electrochemical conditions as for SECCM but using a larger droplet-based cell. The work function and binding energy scale of the spectrometer were calibrated using the Fermi edge and 3d_5/2_ peak recorded from a polycrystalline Ag sample prior to the experiments. Data were analyzed in the CasaXPS package using Shirley backgrounds and mixed Gaussian–Lorentzian (Voigt) line shapes. For compositional analysis, the analyzer transmission function was determined using clean metallic foils to determine the detection efficiency across the full binding energy range.

## Results and Discussion

### Characterization of Locally Anodized Glassy Carbon

Local surface anodization was performed with SECCM in the static mode using a ca. 30 μm diameter pipet filled with 5 mM H_2_SO_4_ by applying a potential of +1.5 V (vs Ag/AgCl QRCE) for 60 s. A typical current–time profile during anodization is shown in Figure S2. A sharp surge in current was recorded at short times from capacitive and Faradaic contributions, and the current then decreased over time until reaching a minimum at about 10 s (ca. 8.4 nA, ∼206 μA cm^–2^ considering the area of the anodized surface obtained by SEM, a spot ca. 72 μm in diameter), with a further steady increase reaching ca. 10.9 nA at 60 s. The total charge transferred during the anodization process was ca. 0.61 μC.

**Figure 2:**
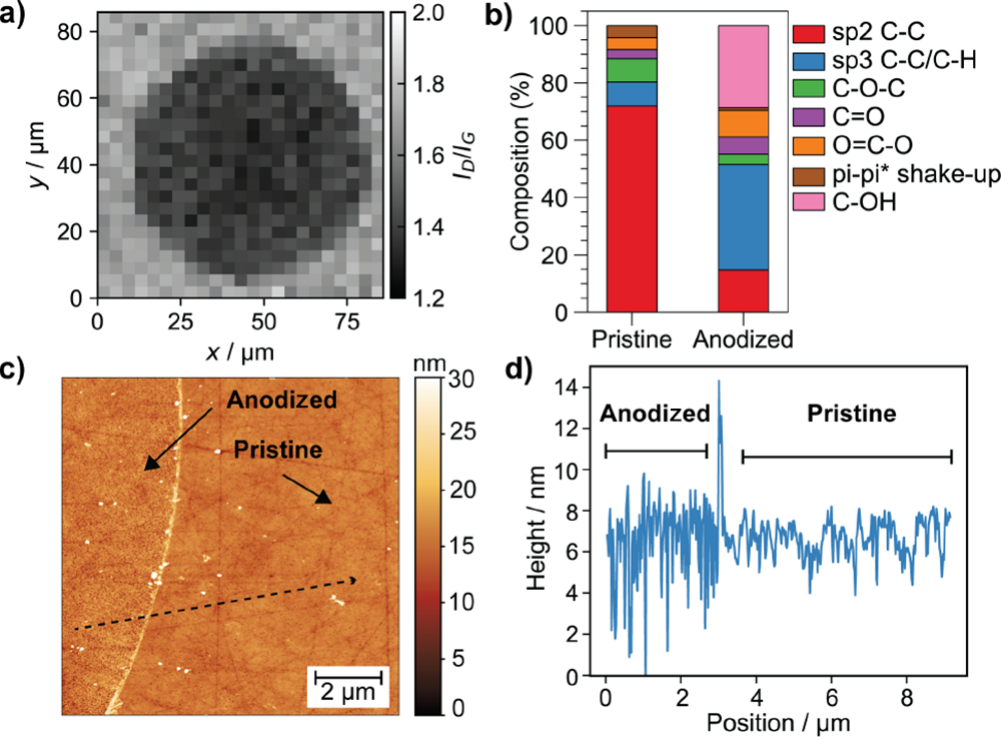
(a) Raman map obtained by plotting the ratio of the D and G bands (*I*_D_/*I*_G_) typical of carbon materials, where the locally anodized area can be readily observed by a slightly smaller *I*_D_/*I*_G_ ratio (illustrated by a darker area in the map). (b) Relative composition (%) of C-based functional groups obtained by analyzing the C 1s XPS spectra on pristine and anodized GC samples. See Figure S6 for high-resolution XPS spectra. (c) AFM image of surface topography covering pristine and anodized regions of the GC electrode. (d) Line scan profile of surface topography extracted from the dashed black arrow indicated in panel c covering anodized and pristine regions. Local anodization was carried out for 60 s in 5 mM H_2_SO_4_ using a ca. 30 μm pipet probe.

The locally anodized GC surface was characterized by Raman microscopy, EDS, XPS, and AFM. Figure S3 shows representative Raman spectra recorded for pristine and anodized regions of the GC. These spectra display the D and G bands typical of carbon-based materials, characteristic of C(sp^3^) and C(sp^2^),^54,55^ at 1349 and 1594 cm^–1^, respectively. Although the spectra recorded in pristine and anodized areas were qualitatively similar, quantitative differences are observed by mapping the intensity ratio of the D and G bands (*I*_D_/*I*_G_) (Figure 2a), which informs on the degree of disorder in carbon materials.^56^ The D/G band ratio was slightly smaller in the anodized region, suggesting a relative decrease of C(sp^3^) compared to C(sp^2^) after anodization and consistent with previous studies using carbon-fiber electrodes.^57^ It is not clear what causes this decrease in the *I*_D_/*I*_G_ ratio, with some hypotheses including the decarboxylation of the carbon surface^57^ or the effect from molecular vibrations’ contribution in the Raman spectra (by functional groups) rather than phonon vibrations by a higher degree of graphitization.^41^ Increased carbon amorphization could also have the same effect in the *I*_D_/*I*_G_ ratio.^54^

Changes in chemical composition on the GC surface after anodization were first evaluated by EDS, as a difference in contrast between anodized and pristine regions was observed by SEM (Figure S4a). A slight increase in oxygen content (from 0.05 ± 0.07 to 0.15 ± 0.05 O at. %) was recorded after anodization (Figure S4b). Note that bulk carbon contributes significantly to these EDX signals, which are also close to the limit of detection. A more accurate quantification of surface chemistry is provided by XPS. Significant differences in chemical composition were obtained by XPS, which provides a higher depth resolution than EDS and highlights the surface changes. XPS survey spectra are shown in Figure S5, whereas high-resolution C 1s and O 1s deconvoluted spectra are shown in Figures S6 and S7, respectively. Table S1 summarizes the assignment of functional groups by binding energy. The oxygen content (in atomic %) increased from 13.8 to 30.2% after anodization, whereas the carbon content decreased from 83.8 to 66.8%. Figure 2b shows the relative content of carbon functional groups between pristine and anodized GC surfaces. The most significant changes from the C 1s spectra (Figure S6) were the strong decrease in C(sp^2^) (from 72 to 14.7%), increase in C(sp^3^) (from 8.3 to 36.9%), and generation of alcohol (C–OH) groups (28.7%) that were not present on the pristine GC. These trends are similar to those found on previous GC anodization studies.^32,36,58^ Other groups such as carboxylates (O=C–O) and carbonyls (C=O) also increased by the anodization, from 4.1 to 9.3% and 3.2 to 5.9%, respectively, whereas the content of ethers/epoxides (C–O–C) decreased from 8.1 to 3.6% and π–π* shake-up, a feature of a delocalized (aromatic) graphitic network,^59^ also decreased from 4.3 to 0.9%. This decrease in graphitic content after anodization is relevant because, as highlighted earlier, DA adsorption has been recently proposed to occur between the π-system of the aryl ring of the catechol and the graphitized regions of the carbon surface, as supported by computational studies^32^ and also observed in previous experiments where the adsorption of electroactive DA occurred efficiently on low-defective graphitic surfaces.^42,43^ Two main regions can be observed in the O 1s spectra (Figure S7): C–O from alcohols, ethers/epoxides, and esters and C=O from ketones, carboxylic acids, and esters. The overall change demonstrates a strong increase in C–O from 27 to 57.4% and decrease in C=O from 59.6 to 37.2% on the anodized GC surface. This observation is likely to be dominated by the extensive generation of C–OH groups on the surface, as detected in the C 1s spectra.

Anodization led to a significant increase of surface roughness as observed by AFM (Figure 2c) and in the roughness profile across the pristine and anodized regions (Figure 2d), with a noticeable development of nanoscale crevices (Figure S8). Surface roughening was estimated quantitatively by calculating the root-mean-square roughness. The roughness changed by a factor of 1.9 from 0.93 ± 0.05 nm (pristine) to 1.78 ± 0.09 nm (anodized). Increased roughness after anodization will affect the double-layer capacitance, which can be related to electrochemical surface area (ECSA) assuming that the specific capacitance is relatively insensitive to surface functionalization.

### Dopamine Adsorption and Oxidation on Locally Anodized Glassy Carbon

SECCM was used to study DA oxidation on pristine and anodized areas (60 s anodization) using the procedure illustrated in Figure 1d. To ensure conditions where the electrochemical response for DA would be dominated by adsorbed DA, a low DA concentration (30 μM) and a fast voltammetric scan rate (47 V s^–1^) were used while allowing time (a period of 2 s and four periods of 1 s) for DA to adsorb on the GC surface before each of five voltammetric measurements were recorded in sequence. A surface-controlled response was confirmed by a scan-rate study (Figure S9). Noting that mass transport in SECCM is relatively fast and quickly attains a steady state^60^ and that, as a rule of thumb, the steady-state mass transport rate is ca. 10% of the equivalent disk microelectrode,^61^, where *D* ∼6 × 10^–6^ cm^2^ s^–1^ is the DA diffusion coefficient,^45^*c* is the bulk DA concentration (30 μM), and *a* is the pipet radius (0.4 μm), there is sufficient flux from the pipet for >1.4 × 10^–10^ mol cm^–2^ (area of meniscus footprint) to adsorb in each adsorption period (2.8 × 10^–10^ mol cm^–2^ in the first period), in excess of the measured values of adsorbed DA on both pristine and anodized surfaces (*vide infra*).

**Figure 3:**
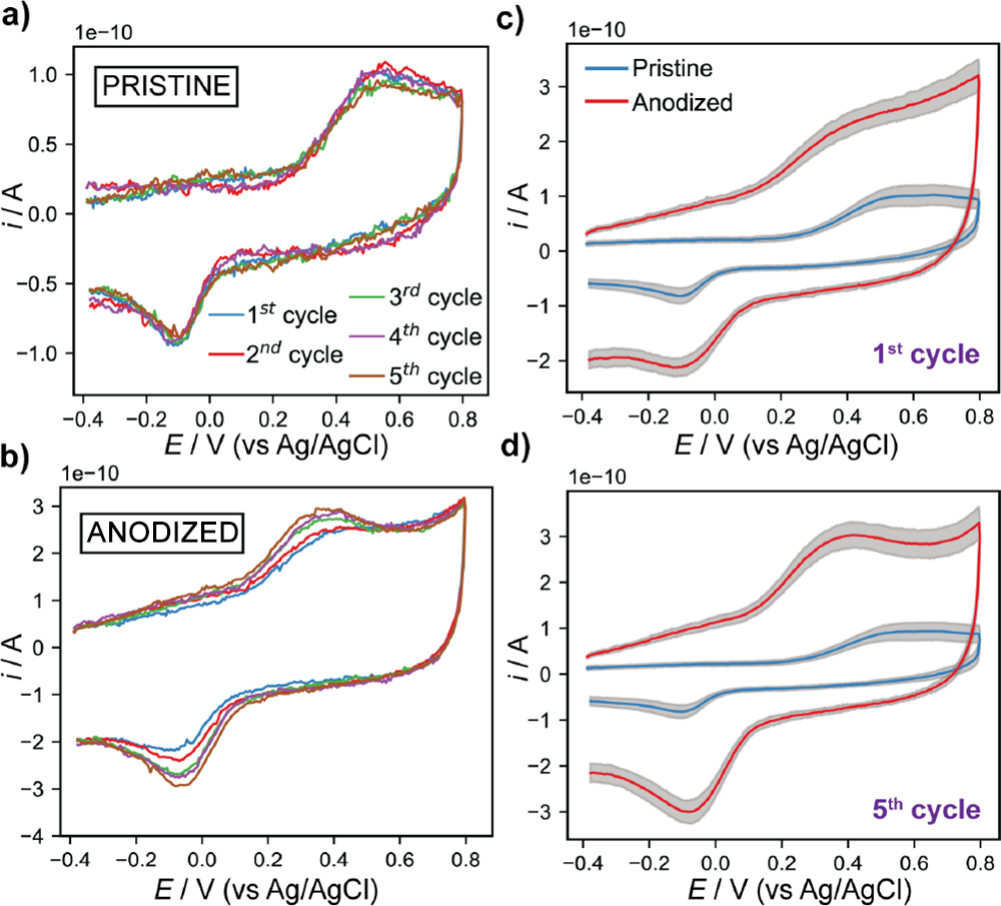
Five consecutive CVs recorded in one location by SECCM where GC was (a) pristine and (b) modified by anodization for 60 s. Average CVs from all the locations of the SECCM measurements where the GC was pristine (blue trace) or anodized for 60 s (red trace) for the (c) first and (d) fifth voltammetric cycles. Gray areas illustrate the standard deviation. A total of 91 and 19 independent measurements were recorded in pristine and anodized areas, respectively. DA (30 μM) in PBS was used in all cases, with 47 V s^–1^ as the scan rate and using a pipet probe of ca. 800 nm diameter.

The pipet probe was translated across both pristine and anodized regions in the same SECCM experiment as illustrated in Figure 1d. Representative SECCM CVs for DA oxidation (forward sweep) and reduction of the product DAQ (reverse sweep) on pristine and anodized regions of GC are shown in Figure 3. Quantitatively similar peak currents were obtained for the five cycles on the pristine GC surface (Figure 3a). This response indicates that DA adsorption to completion takes place quickly on pristine areas before the first cycle, as the oxidation current does not increase upon cycling. Anodic peak currents ca. 65 pA were measured on pristine regions, which are significantly higher than the several pA estimated for a transient diffusion-controlled process (assuming planar diffusion, given that the diffusion layer thickness will be on the micrometer scale at this voltammetric scan rate) or the ca. 1 pA steady-state SECCM current.^62,63^ This confirms that the oxidation and reduction signatures are mainly due to adsorbed DA/DAQ. A different behavior was found for the SECCM CVs obtained on anodized GC (Figure 3b). The measurable increase in DA oxidation peak currents with cycling indicates that DA coverage grows on anodized areas during the experiment. The longer time scale for DA adsorption in anodized GC areas might be a consequence of the larger specific surface area and less accessible nanoscale surface features, as observed on AFM images (*vide supra*), especially relevant at low DA concentrations as used herein. DA adsorption has also been reported to increase for several minutes following the fracture of GC surfaces.^31^ The nature of SECCM, where measurements are carried out quickly after contact with a fresh surface, facilitates the visualization of such time-dependent phenomena. Figure 3c,d shows comparatively the average CVs for all the SECCM measurements on pristine and anodized areas (first and fifth cycles). These plots provide a clear view of the different electrochemical response for DA adsorption and electro-oxidation observed on the distinct GC regions. The significant increase in capacitive current on anodized areas is consistent with the roughening of the GC surface structure as detected by AFM and as previously reported in GC anodization studies.^41,64^ Further analysis is required (*vide infra*) to unequivocally elucidate the relationship between the surface structure and DA adsorption, taking into account the effect of the increased surface area.

**Figure 4:**
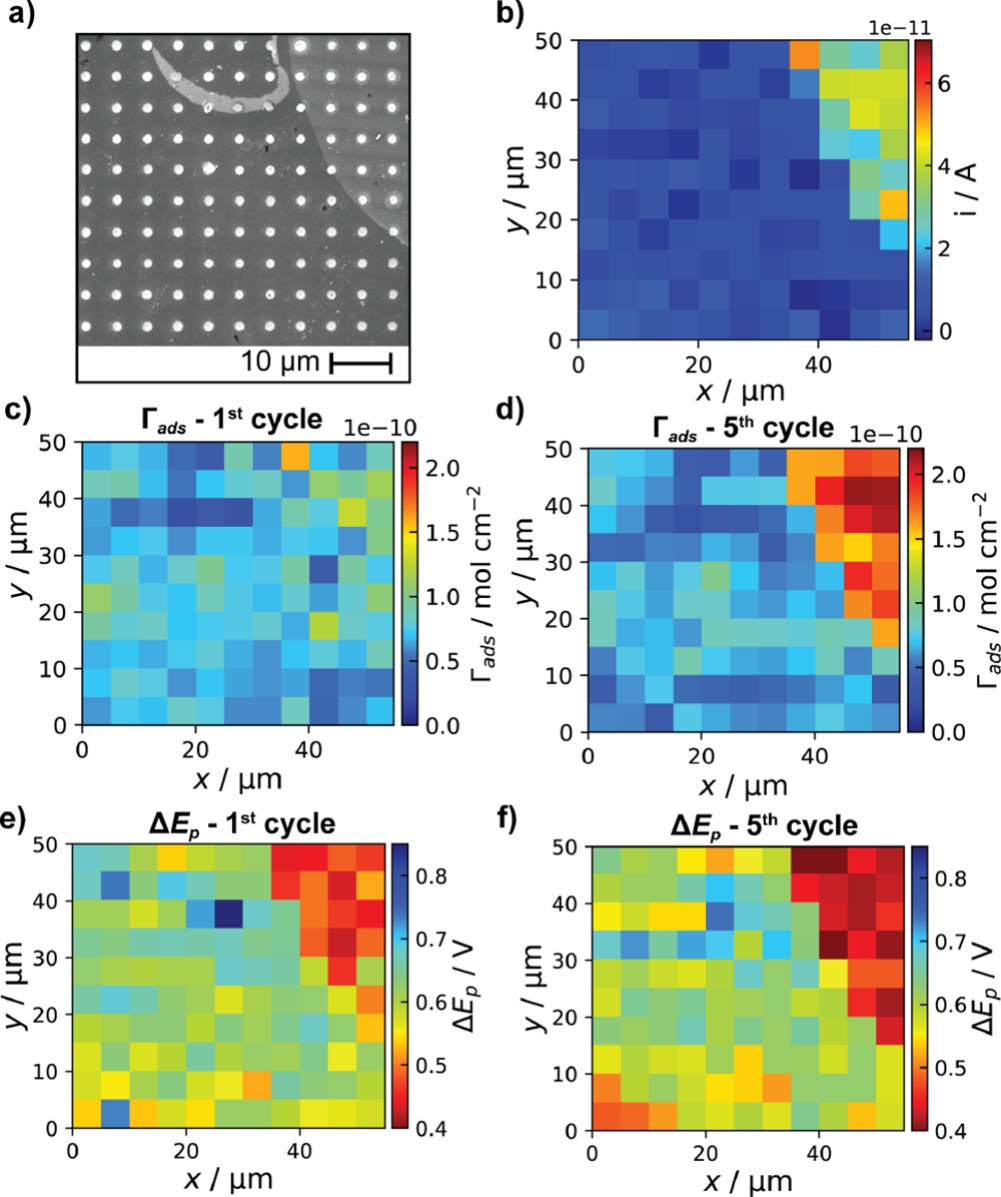
(a) SEM image of the SECCM scan area covering pristine (darker) and anodized for 60 s (brighter) regions of the GC electrode. (b) Spatially resolved map of *i* obtained at +0.3092 V vs Ag/AgCl QRCE (first cycle) showing DA oxidation activity across the GC surface. Baseline was corrected to remove the effect of the different capacitive currents. (c, d) Spatially resolved maps of DA surface concentration (Γ_ads_) for the first and fifth voltammetric cycle, respectively. (e, f) Spatially resolved maps of the peak potential difference between DA oxidation and DAQ reduction peaks (Δ*E*_p_) for the first and fifth voltammetric cycle, respectively. SECCM maps contained 110 pixels (55 × 50 μm^2^ with 5 μm as the hopping distance).

**Figure 5:**
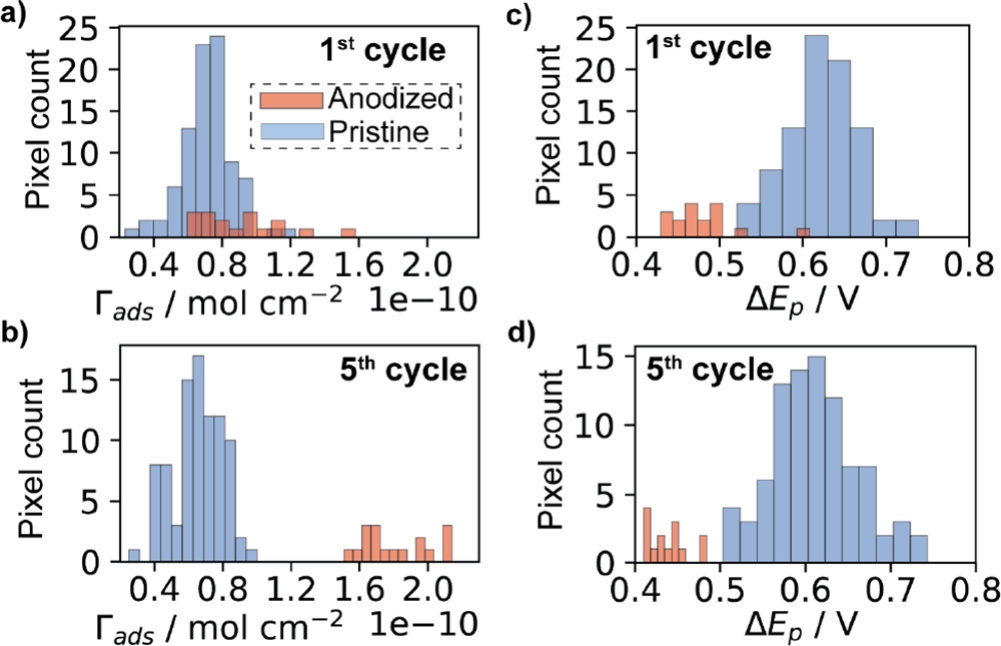
Histograms of DA surface concentration (Γ_ads_) calculated for the (a) first and (b) fifth voltammetric cycles. Histograms of Δ*E*_p_ for the (c) first and (d) fifth voltammetric cycles. Data were obtained from anodized (red bars) and pristine (blue bars) regions of the SECCM scan illustrated in Figure 4.

Spatially resolved electrochemical (*i*–*E*) movies can be constructed from the data to represent visually the effect of the locally anodized GC surface on DA reactivity. Movie S1 represents the *i*–*E* response of the first voltammetric cycle where different local electrochemistry on pristine and anodized areas is obtained, which can be correlated to their corresponding location in the SEM images (Figure 4a). The voltammetric baseline was corrected to remove the effect of the capacitive current, highlighting only the information contained in the DA oxidation peak. Figure 4b shows a frame of the SECCM movie representing the current at ca. +0.31 V (near the onset of dopamine oxidation), where it can be seen that larger currents were obtained on anodized areas. However, there was not a clear difference in the magnitude of the DA oxidation peak currents between pristine and anodized regions for the first voltammetric cycle. This fact is highlighted through a spatially resolved map of the electroactive DA surface concentration (Γ_ads_) shown in Figure 4c, with Γ_ads_ calculated from *Q* = *nFA*Γ_ads_ after integrating the charge under the DA oxidation peak (*Q*), with *F* the Faraday constant, *n* the number of electrons (2 e^–^), and *A* the electrode area (defined by the *geometric* size of the SECCM droplet footprints; Figure 4a). There appears to be little visual difference in Γ_ads_ between pristine and anodized areas, with much of the anodized data contained within the population distribution of the pristine area (see the Γ_ads_ histogram in Figure 5a). However, a closer inspection revealed that Γ_ads_ was statistically higher on anodized GC (unpaired *t* test, *p* = 0.00001): average values were 71 ± 15 and 91 ± 25 pmol cm^–2^ for the pristine and anodized surfaces, which indicate that the adsorption kinetics is fast and there is a strong transport-controlled component (based on the estimated SECCM flux, *vide supra*), as established for other carbon surfaces.^23^ The fractional surface coverage corresponds to 28 and 36% of the geometric GC surface, respectively. These values are calculated considering ca. 255 pmol cm^–2^ as the theoretical limit for the adsorption of a monolayer of DA in a flat configuration (molecular area: 6.5 × 10^–15^ cm^2^).^65,66^ Larger monolayer coverage values result from DA adsorption in a vertical orientation,^66^ but this occurs at higher adsorbate concentrations for catechol-like molecules.^67^ Previous values reported for DA surface concentrations are of the same order, between 10 and 400 pmol cm^–2^, with the largest values observed for mechanically or electrochemically pretreated GC surfaces,^31^ but are recorded under different conditions from our experiments. It is worth noting that the active surface area is comparatively larger in anodized regions, as inferred by the AFM roughness measurements and capacitive current analysis, and so the fractional DA surface coverage (in terms of specific surface area or ECSA) is significantly smaller in the anodized areas (*vide infra*).

Both the electrochemical movie (Movie S2) and the Γ_ads_ map for the fifth voltammetric cycle (Figure 4d) show that DA adsorption increased in the anodized areas throughout the meniscus contact time compared to little change in the pristine areas. Indeed, a significant change in the population analysis is detected (Figure 5b), with average values being 65 ± 14 and 180 ± 20 pmol cm^–2^ for pristine and anodized areas (i.e., a coverage of 26 and 71% of the geometric GC surface), respectively. Thus, DA takes a longer time to adsorb on all available sites on the anodized surface (noting that there is sufficient flux for adsorption not to be limited solely by mass transport from the pipet in this case, *vide supra*).

### Effect of Anodization on Electron-Transfer Kinetics

**Figure 6:**
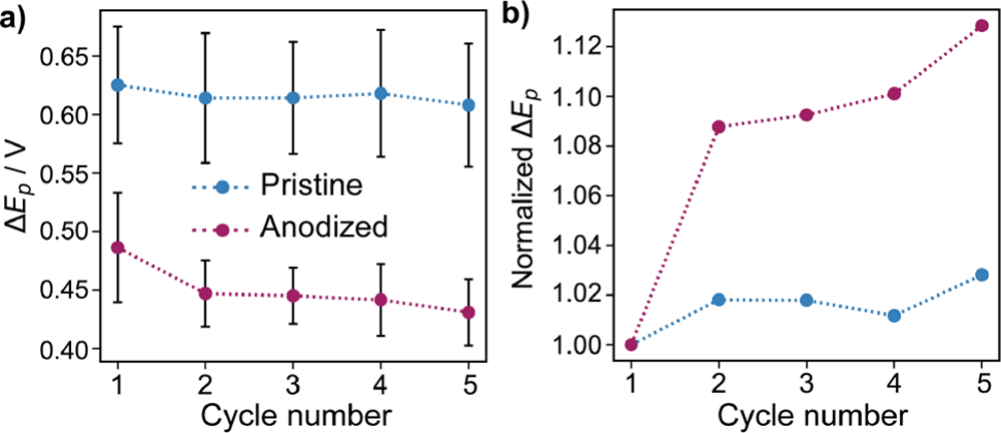
(a) Absolute variation of Δ*E*_p_ with cycle number in pristine (blue trace) and anodized (red trace) locations of the GC surface. (b) Variation of Δ*E*_p_ with cycle number normalized by the value for the first cycle. Higher values in panel b indicate enhanced kinetics related to those recorded in the first cycle. Error bars indicate the standard deviation from all the SECCM pixels for specific locations (pristine or anodized).

To further evaluate the effect of GC anodization on DA electron-transfer kinetics, SECCM maps representing the peak potential difference (Δ*E*_p_) for DA oxidation and DAQ reduction in the first and fifth voltammetric cycles are shown in Figure 4e,f. A notable difference in Δ*E*_p_ values was obtained with DA/DAQ reactions being significantly faster on anodized areas, as also illustrated by analyzing the Δ*E*_p_ histograms (Figure 5c,d). Average Δ*E*_p_ values for the first voltammetric cycle were 0.63 ± 0.05 V (pristine), *cf.* 0.49 ± 0.05 V (anodized) GC. This enhancement in electron-transfer kinetics (smaller Δ*E*_p_)^68^ is consistent with previous studies using anodized GC.^31,32^ For the fifth voltammetric cycle, average values of Δ*E*_p_ were 0.61 ± 0.05 (pristine), *cf.* 0.43 ± 0.03 V (anodized), indicating a slight increase in electron transfer kinetics for anodized GC upon cycling. The change in average Δ*E*_p_ with cycling was further analyzed in terms of absolute values (Figure 6a) and normalized by the value at the first cycle (Figure 6b). While the effect of cycling was negligible in the pristine areas (where the adsorbed quantity of DA was also constant), in anodized areas, the kinetics was statistically enhanced (unpaired *t* test, *p* = 0.0003) and is correlated with increased DA coverage, consistent with a self-catalytic effect.^69^

### Effect of the Electrochemical Surface Area on the Apparent Dopamine Adsorption

Γ_ads_ values are calculated using the geometric electrode area, but the increased surface area (observed by AFM) or actual ECSA needs to be considered. The capacitive charge (*Q*_c_) is higher on anodized areas as shown in the SECCM map of *Q*_c_ in Figure S10 (where the integrated charge was between −0.375 and −0.275 V to avoid the DA oxidation process; Figure S11). Table S2 summarizes the average values of *Q*_c_ for anodized and pristine GC and the corresponding anodized/pristine ratio after subtracting the contribution from stray capacitance. The anodized/pristine *Q*_c_ ratio was 3.3 for the first voltammetric cycle, which is larger than the surface roughness ratio calculated by AFM (ca. 1.9). The value obtained from AFM will always underestimate the true area due to the finite tip size that restricts access to small and deep features of the rough anodized surface. Yet, the *Q*_c_ ratio is only ca. 50% larger than the AFM surface roughness measurement, suggesting that functional groups on the carbon surface after anodization only make a minor contribution to the specific capacitance in this potential range. We thus propose that the *Q*_c_ ratio is a reasonable estimate of the ECSA enhancement upon anodization. Certainly, any functionalization of the GC upon anodization does not result in any change in wetting, as the SECCM droplet footprints are very consistent throughout both areas of the GC surface (Figure S12).

We evaluated any correlation of *Q*_c_ and Γ_ads_ with cycle number for pristine and anodized areas of GC. In pristine areas (Figure S13a–c), there is only a slight variation in both *Q*_c_ and Γ_ads_, likely from typical random experimental variation. In contrast, a small but clear increment in *Q*_c_ with cycling is observed in anodized regions (Figure S13d), alongside a significant increase of Γ_ads_ (Figure S13e). By comparing the evolution of both parameters (normalized to the first cycle, Figure S13f), the enhancement in DA surface concentration is unambiguously higher than that for *Q*_c_. For instance, *Q*_c_ and Γ_ads_ increased by ca. 1.2× and 2.0× up to the fifth cycle, respectively. The amount of electroactive DA adsorbed thus genuinely increases with cycling on anodized GC, indicating finite adsorption kinetics, probably associated with DA transport to reach all the available electrode active sites on the nanostructured surface.

**Figure 7:**
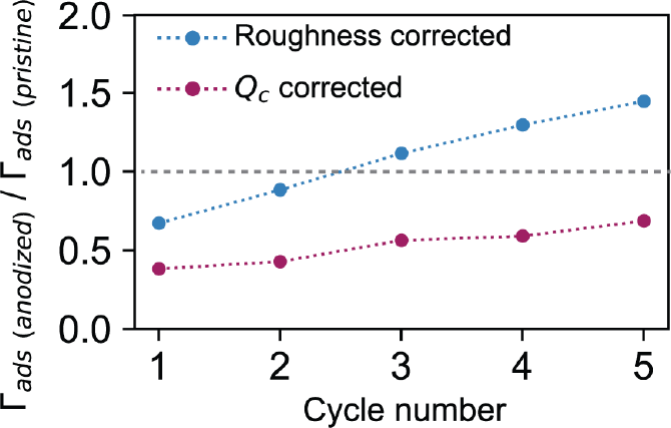
Variation of the ratio between Γ_ads_ in anodized and pristine GC as a function of voltammetric cycle number corrected by the surface roughness ratio (blue trace) and the *Q*_c_ ratio (red trace).

We now consider the relative DA surface concentration on anodized vs pristine areas, , accounting for specific surface area, by using both the anodized/pristine ratio in *Q*_c_ (ECSA) and surface roughness for normalization (Figure 7). After correcting by the *Q*_c_ ratio, the DA surface concentration for anodized GC is actually lower than that for pristine GC. For the early cycles, the mass transport rate will contribute to this observation, but limited mass transport from the SECCM pipet will not be a major factor by the fifth cycle.

The surface roughness (AFM) normalization produces a slightly higher relative DA surface concentration on anodized vs pristine GC (after the third cycle), but it is clear from this analysis that anodization and functionalization of the GC surface do not lead to significantly enhanced DA specific adsorption and probably decrease the amount adsorbed based on the specific surface area of anodized GC.

### Effect of Anodization Time on Dopamine Adsorption and Reactivity

**Figure 8:**
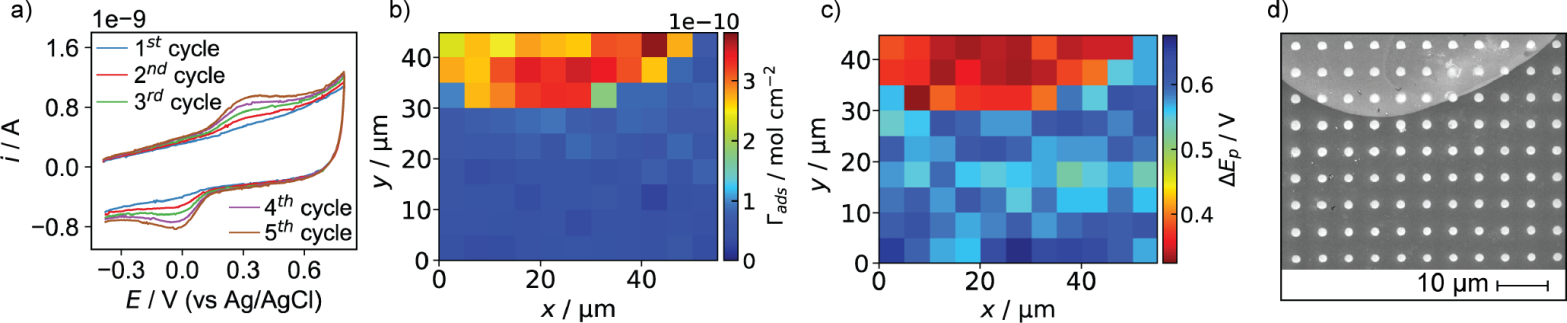
(a) CVs (five cycles) obtained from one location of the SECCM measurement where anodization was carried out for 300 s. (b) Spatially resolved map of DA surface concentration (Γ_ads_). (c) Spatially resolved map of the peak potential difference between DA oxidation and DAQ reduction peaks (Δ*E*_p_). (d) SEM image of the SECCM scan area covering pristine (darker) and anodized for 300 s (brighter) regions. Maps contained 99 pixels (55 × 45 μm^2^ with 5 μm as the hopping distance).

To further test whether surface anodization decreased the adsorption efficiency of electroactive DA, the anodization time was increased up to 300 s (*i*–*t* curve in Figure S14, yielding a total charge of 4.1 μC, about 7× more than for 60 s anodization). Figure 8a shows five consecutive voltammetric cycles for this GC surface, for which it can be seen that (1) capacitive currents are significantly increased (ca. 90 pA at −0.375 V vs Ag/AgCl QRCE) compared to those recorded after anodization for 60 s (ca. 35 pA), indicating that the ECSA has been further increased; (2) relatively small DA oxidation and DAQ reduction currents are obtained for the first cycle; and (3) DA oxidation and DAQ reduction currents increased upon cycling, suggesting again that DA requires longer times to adsorb on anodized regions. The spatially resolved SECCM experiments highlight these differences for the electrochemistry of DA behavior in pristine and anodized areas, as presented in Figure 8b,c where maps of Γ_ads_ and Δ*E*_p_ for the fifth cycle are shown and correlated to the identical location SEM image (Figure 8d). By further analyzing Γ_ads_, *Q*_c_ ratio (as a measure of ECSA enhancement), and Δ*E*_p_ (kinetics), the previous findings are further supported: surface anodization increases the DA electron-transfer kinetics but lowers DA adsorption compared to pristine GC when ECSA is accounted for. Thus, although the average Γ_ads_ (for the fifth cycle) was 300 ± 37 pmol cm^–2^ on anodized areas compared to 58 ± 17 pmol cm^–2^ on pristine areas, the *Q*_c_ ratio between anodized and pristine surface was ca. 11.2 (corrected for stray capacitance), and accounting for ECSA means that Γ_ads_ on the anodized surface is about half of that found on pristine GC. This was significantly smaller than that found for 60 s anodization, which was about 69% of that found on pristine GC (fifth cycle). The DA flux from the pipet (*vide supra*) obviously becomes a more important factor for the rougher surface.

Faster electron-transfer kinetics was also observed on GC regions anodized for 300 s, with even smaller values of Δ*E*_p_ for DA electrochemistry when the anodization time was longer: 0.36 ± 0.02 V (300 s anodization) compared to 0.43 ± 0.03 V for 60 s anodization (fifth voltammetric cycle). EDS measurements detected a higher oxygen content (0.6 ± 0.1%) for 300 s anodization compared to 0.05 ± 0.07% for pristine and 0.15 ± 0.05% for 30 s anodized areas (Figure S15). Interestingly, DA surface concentration (normalized by ECSA increment) upon cycling (Figure S16) increased at a similar rate for 60 and 300 s anodization (4.5 ± 0.4 and 4.9 ± 0.3 pmol cm^–2^ s^–1^, respectively). This fact suggests that the normalized DA adsorption rate (in the period from cycle 1 to cycle 5) is similar for both anodized surfaces and the differences might be mainly due to an onset time required until DA effectively reaches all active sites in rougher surfaces, with this onset time being shorter on less rough surfaces.

## Conclusions

SECCM with complementary co-located surface characterization has revealed how changes in the surface structure of a GC electrode after anodization in acid affect DA adsorption and electrochemistry. Static-mode SECCM was deployed to perform local surface anodization on a GC surface, which was subsequently probed by spatially resolved voltammetric SECCM to characterize both pristine and anodized regions within the same electrochemical experiment. A fast scan rate and micromolar concentrations of DA were used to work under conditions where adsorbed DA dominates the electrochemical kinetics, revealing that both DA adsorption and electrochemical kinetics were clearly affected by GC anodization.

Anodization creates a significantly rougher and nanostructured electrode than that found on pristine surfaces. This structural change appeared to increase the time required for DA to adsorb on available surface sites. Together with the increase in surface roughness detected by AFM, anodized regions also show significantly higher ECSAs. Normalizing the DA surface concentration by ECSA enhancement clearly shows that anodization decreases the DA adsorption efficiency (i.e., actual surface coverage) for the same time scale and conditions than on pristine GC regions. The lower coverage at early times is, at least in part, due to limited DA flux from the pipet. However, at longer times, there are kinetic effects that might be associated with the restricted accessibility of some adsorption sites. The overall lower cover of DA normalized by ECSA is consistent with a lower graphitic carbon on the anodized GC surface, where electroactive DA adsorption seems to take place.^11,32,43^

DA electron-transfer kinetics was notably enhanced on anodized GC regions, with ΔE_p_ values decreasing with increasing times of anodization (60 vs 300 s). It is tempting to attribute enhanced electron-transfer kinetics to a different interaction of DA with oxygenated groups created on the GC surface, as a higher content of oxygen moieties is detected at longer anodization times, although there is still a proportion of the surface that is graphitic according to XPS data. Increasing surface roughness in carbon materials without changes in surface chemistry can also lead to apparent faster electron-transfer kinetics.^70^ A DA self-catalytic effect is also observed on anodized GC as there was a decrease in ΔE_p_ values with increasing DA surface concentration, thus confirming that DA self-catalysis also occurs between adsorbed DA molecules and not only between adsorbed and solution-based molecules.^69^

In conclusion, this study reveals how changes in the GC surface nanostructure after anodization in acidic media control relevant phenomena of DA reactivity on carbon surfaces: adsorption and electrochemical kinetics. These results highlight the importance of considering factors such as changes in ECSA or adsorption rates when comparing DA adsorption in carbon electrodes with different roughness or porosity, as obtained after anodization. Overall, this study also demonstrates the versatility of SECCM for local modification of electrode surfaces and for resolving surface structure–activity relationships in heterogeneous materials.

## Supporting Information Available

Additional electrochemical data and XPS and EDS spectra (PDF)
Spatially resolved electrochemical (*i*–*E*) movie (110 pixels over a 55 × 50 μm scan area, hopping distance = 5 μm) obtained with the SECCM protocol (first voltammetric cycle), visualizing the activity of DA oxidation on a GC surface locally anodized for 60 s. The pipet probe contained 30 μM DA in PBS, and the scan rate was 47 V s^–1^ (MP4)
Spatially resolved electrochemical (*i*–*E*) movie (110 pixels over a 55 × 50 μm scan area, hopping distance = 5 μm) obtained with the SECCM protocol (fifth voltammetric cycle), visualizing the activity of DA oxidation on a GC surface locally anodized for 60 s. The pipet probe contained 30 μM DA in PBS, and the scan rate was 47 V s^–1^ (MP4)
