# Supplementary material for: Surface Nanostructure Effects on Dopamine Adsorption and Electrochemistry on Glassy Carbon Electrodes
Source: J Phys Chem C Nanomater Interfaces. 2022 Jul 29;126(31):13399–408. doi: 10.1021/acs.jpcc.2c02801 (PMC9377355; doi:10.1021/acs.jpcc.2c02801)
Supplement: Supplementary file 1 — jp2c02801_si_001.pdf [file jp2c02801_si_001.pdf]

SUPPORTING INFORMATION

Surface Nanostructure Effects on Dopamine Adsorption and Electrochemistry on Glassy Carbon Electrodes

Dalia L. Swinya,[1] Daniel Martín-Yerga,[1]* Marc Walker,[2] Patrick R. Unwin[1]*

[1]*Department of Chemistry, University of Warwick, Coventry CV47AL, United Kingdom*

[2]*Department of Physics, University of Warwick, Coventry CV47AL, United Kingdom*

*Corresponding authors e-mail:

daniel.martin-yerga@warwick.ac.uk (D.M-Y)

p.r.unwin@warwick.ac.uk (P.R.U)

SUPPORTING FIGURES

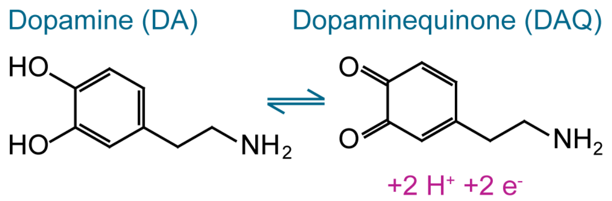

Figure S1. Dopamine (DA) undergoes a 2-electron 2-proton electro-oxidation process to dopaminequinone (DAQ).

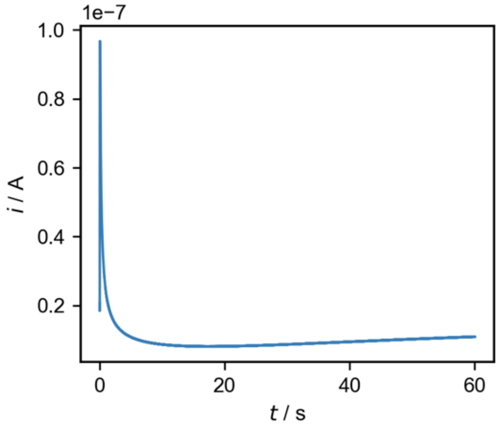

Figure S2. Chronoamperometric (*i-t*) curve obtained during the local anodization of a glassy carbon (GC) surface at +1.5 V (vs Ag/AgCl quasi-reference/counter electrode (QRCE)) for 60 s using static-mode scanning electrochemical cell microscopy (SECCM). The pipet probe had a ca. 30 μm diameter and was filled with a 5 mM H_2SO_4 solution.

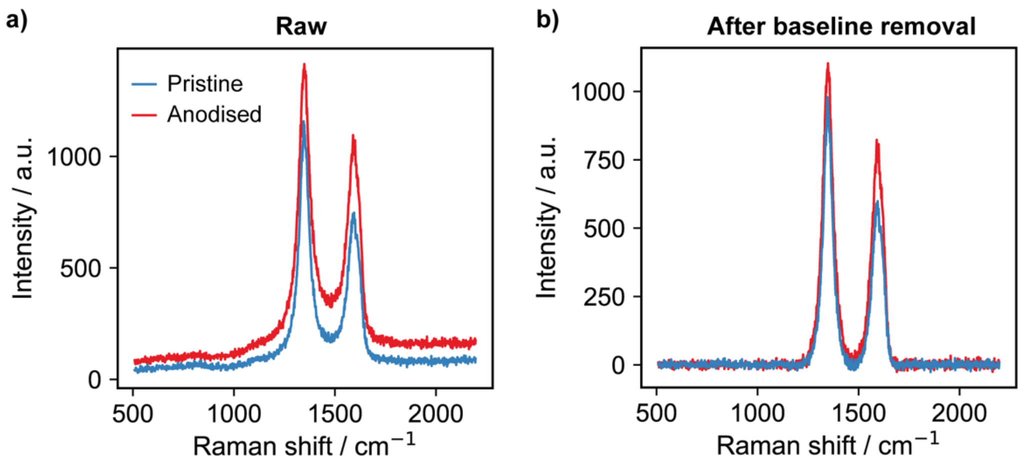

Figure S3. (a) Representative Raman spectra obtained in pristine and anodized regions of a GC surface. (b) Same spectra after baseline removal to highlight the differences in peak intensities.

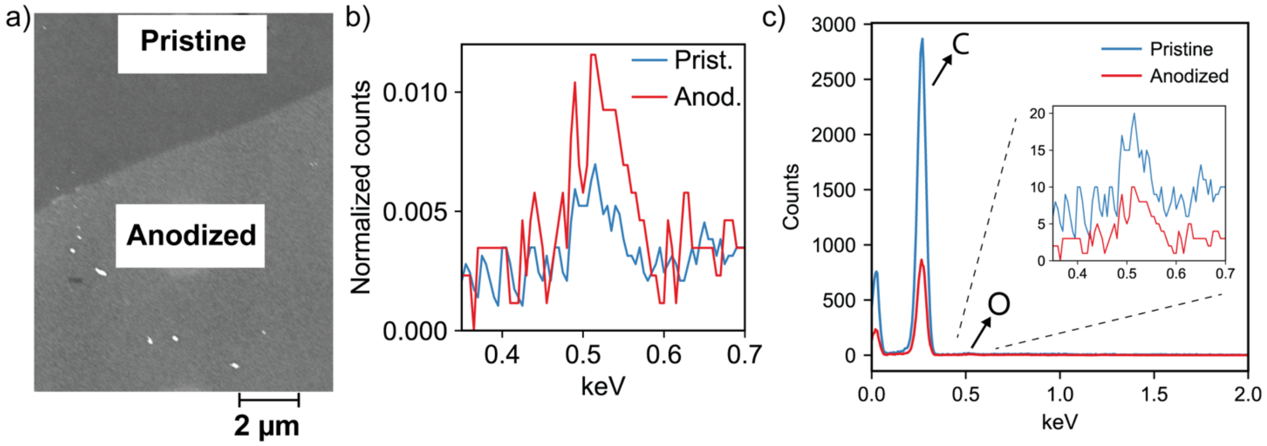

Figure S4. (a) Scanning electron microscopy (SEM) image of the GC surface showing a boundary between the anodized (brighter) and pristine (darker). (b) Energy-dispersive X-ray spectroscopy (EDS) spectra obtained from anodized and pristine regions. EDS spectra shows the O region after normalization of the C signals to highlight the changes in O/C ratio. Raw EDS spectra is shown in (c).

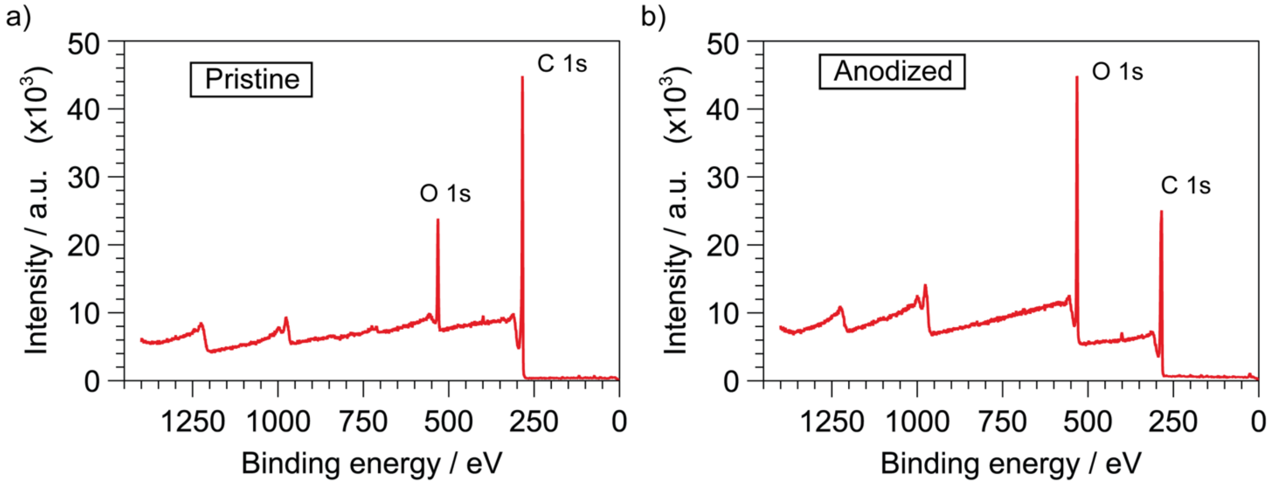

Figure S5. X-ray Photoelectron Spectroscopy (XPS) survey spectra for (a) pristine and (b) anodized GC surfaces.

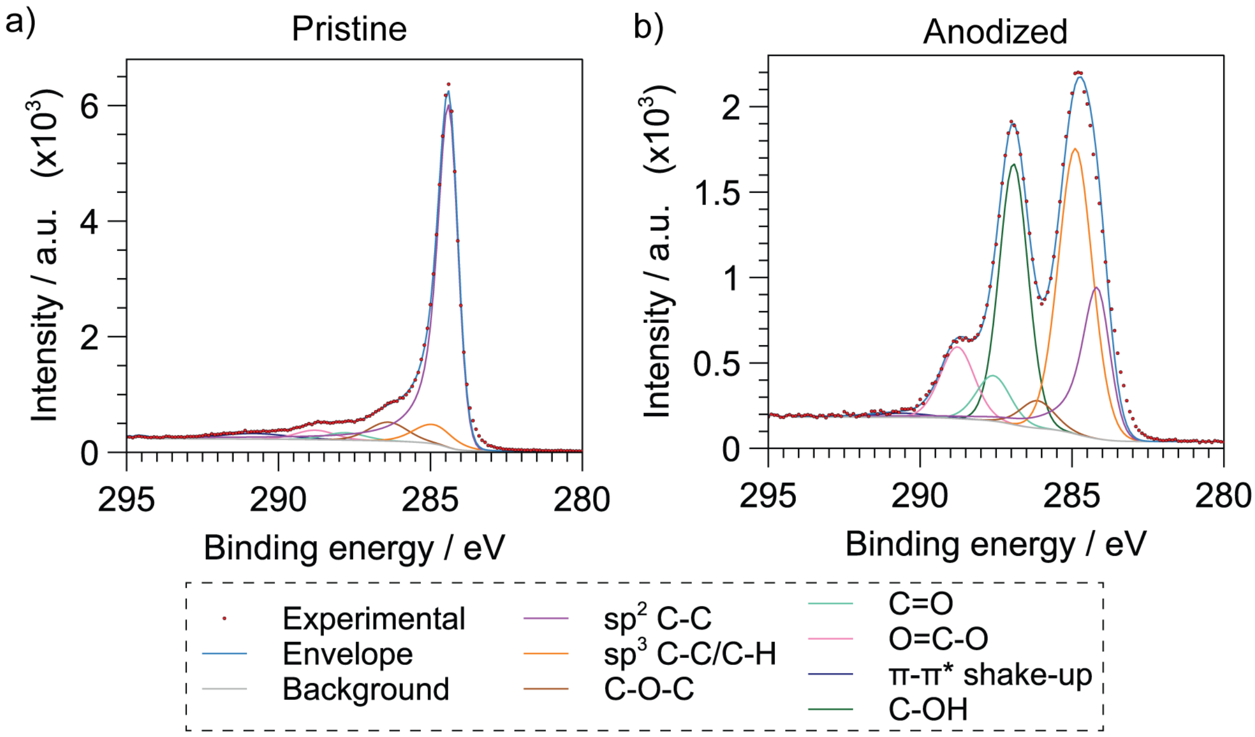

Figure S6. High-resolution C 1s spectra of (a) pristine and (b) anodized GC surfaces, with fitted deconvolution. Table S1 summarizes the assignment of functional groups by binding energy.

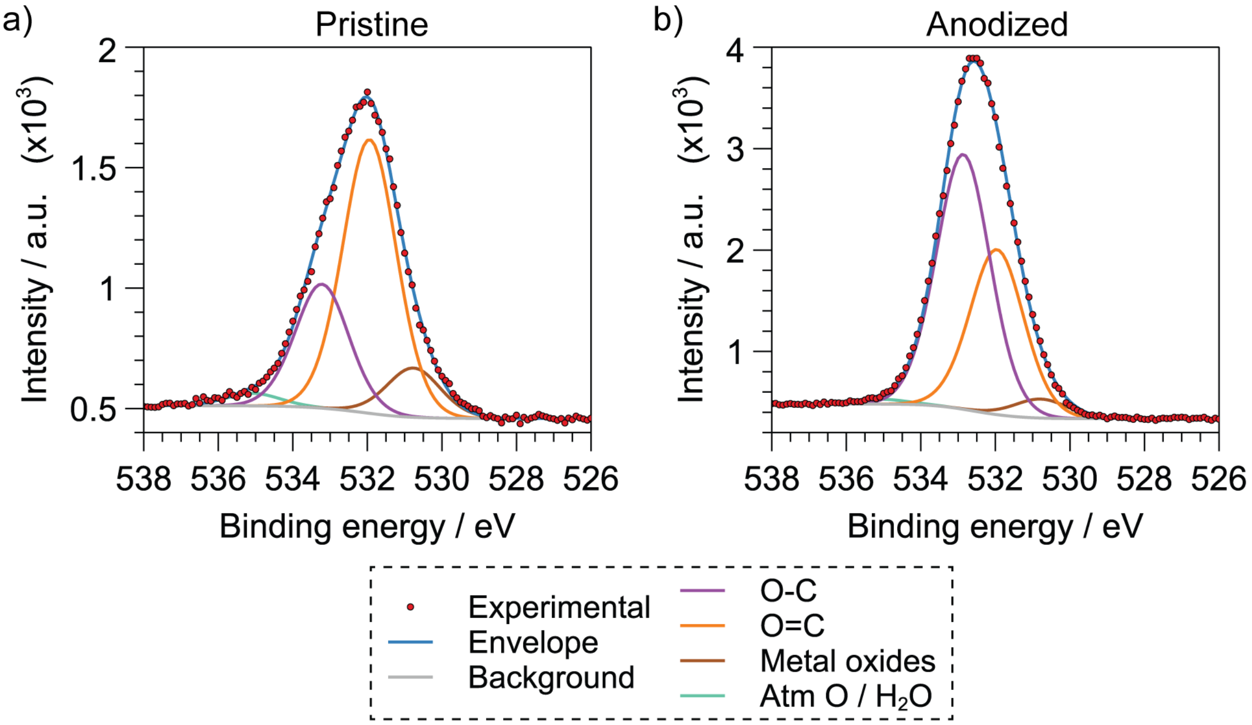

Figure S7. High-resolution O 1s spectra of (a) pristine and (b) anodized GC surfaces, with fitted deconvolution. Table S1 summarizes the assignment of functional groups by binding energy.

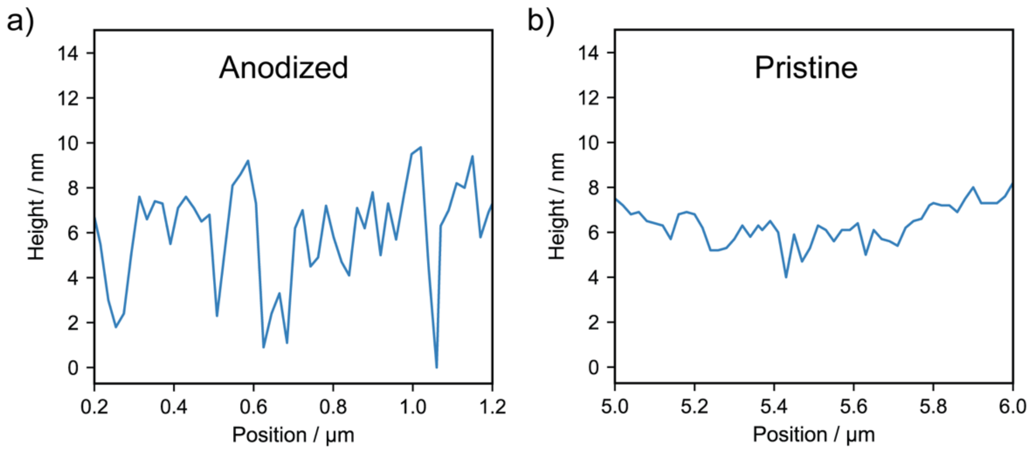

Figure S8. Zoomed-in regions of the line scan profiles of surface topography extracted by atomic force microscopy (AFM) imaging from (a) anodized and (b) pristine regions.

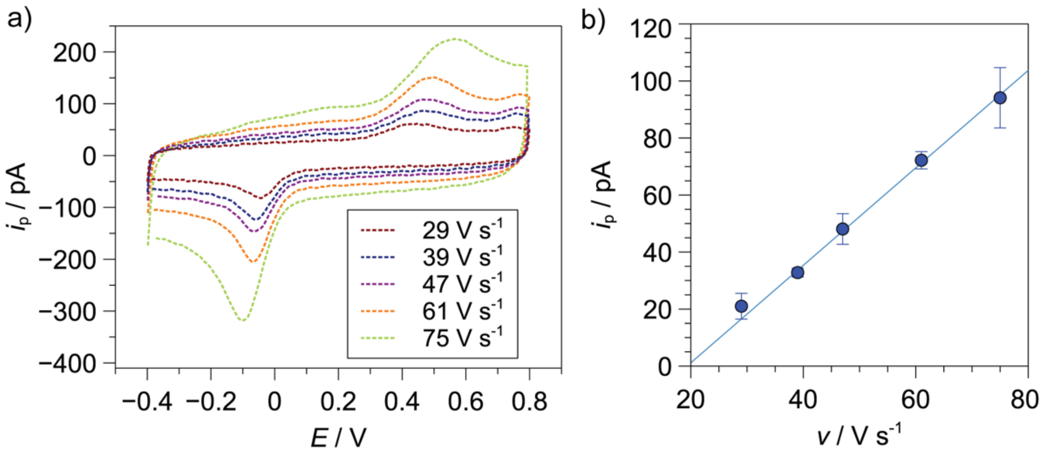

Figure S9. (a) Voltammetric response for 30 µM DA in PBS at different scan rates (29, 39, 47, 61 and 75 V s^{-1}) recorded by SECCM at different locations of a pristine GC surface. DA was allowed to adsorb for 2 s before each voltammetric measurement. A pipet probe of ca. 800 nm diameter was used. (b) Relationship between oxidative peak currents and scan rate. Error bars represent the standard deviation from three independent measurements at different GC locations, and the solid line represents the fitted linear regression curve ($R^2 = 0.997$). A linear relationship between oxidative peak currents and scan rate demonstrates a surface-controlled dopamine oxidation reaction.

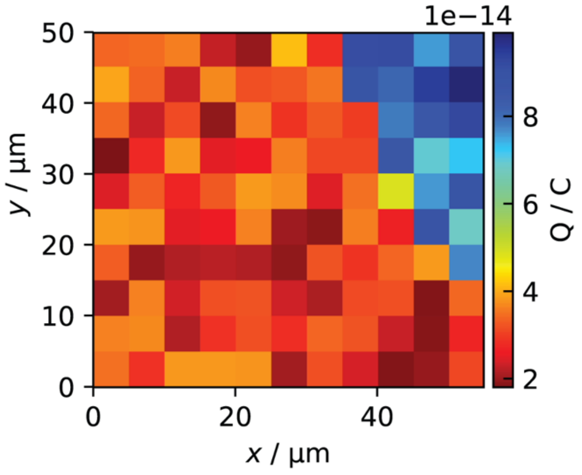

Figure S10. Spatially-resolved map obtained by integrating the charge (Q) between -0.375 and -0.275 V vs Ag/AgCl QRCE (1st voltammetric cycle). This map shows mostly the capacitive contribution from the electrochemical response well before the dopamine (DA) oxidation process. SECCM maps contained 110 pixels (55x50 μm^2 with 5 µm as hopping distance). The pipet probe contained 30 µM DA in a phosphate buffer saline (PBS) solution, and voltammetric scan rate was 47 V s^{-1}.

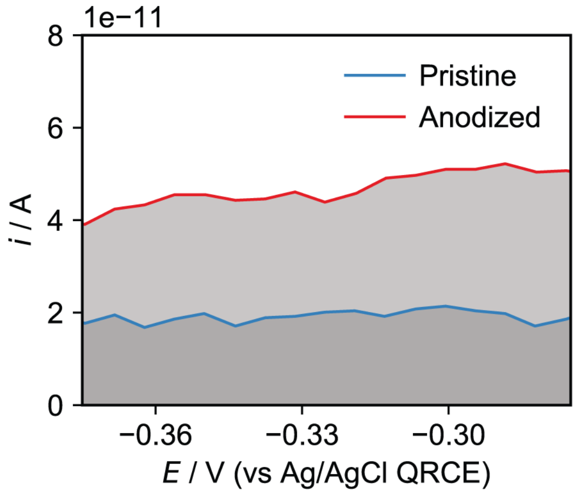

Figure S11. Region of the cyclic voltammetry where the capacitive charge (Q_c) was integrated to estimate the enhancement of electrochemically active area on anodized (red trace) compared to pristine (blue trace) regions of the GC surface.

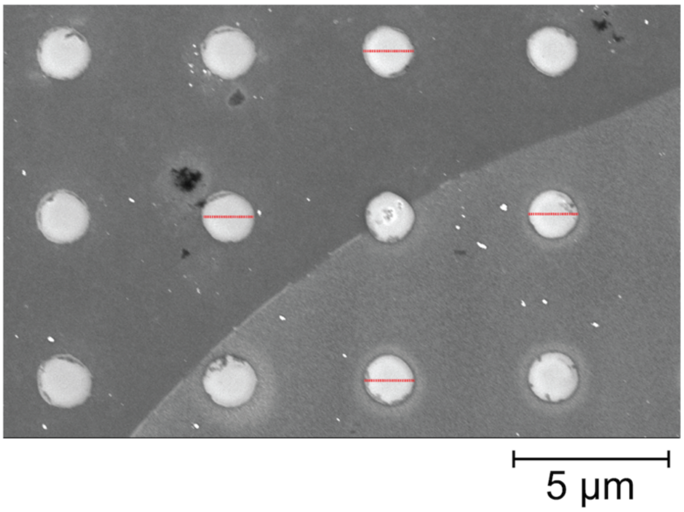

Figure S12. Scanning electron microscopy (SEM) image of SECCM footprints on pristine (darker area) and anodized (brighter area) regions of a GC surface locally anodized for 60 s. Electrolyte footprints were of similar size (ca. 1.6 µm diameter, red lines) on both pristine and anodized regions, indicating that hydrophilicity (i.e., wetting) was similar in both regions.

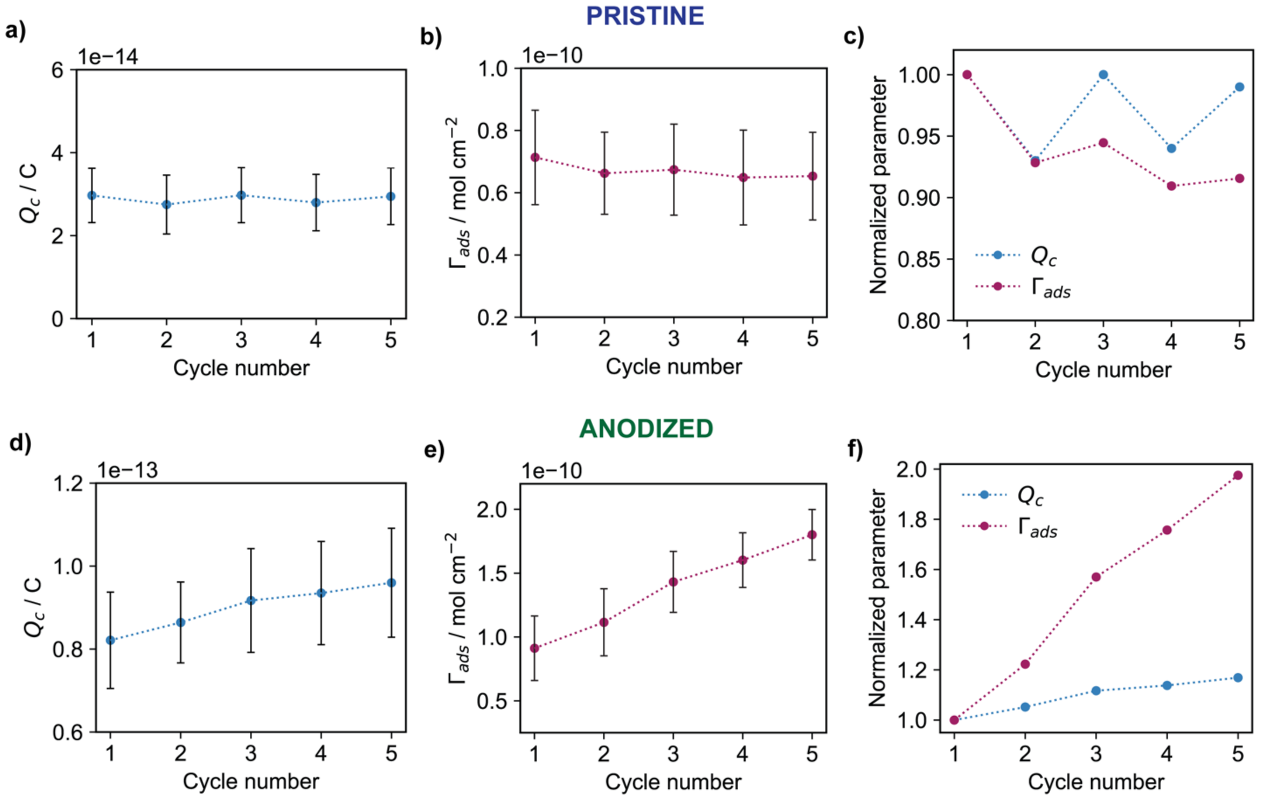

Figure S13. Absolute variation of (a) Q_c and (b) Γ_{ads} with cycle number for pristine GC. (c) Variation of Q_c (blue trace) and Γ_{ads} (red trace) with cycle number normalized by the value at the 1st cycle in pristine GC regions. Absolute variation of (d) Q_c and (e) Γ_{ads} with cycle number in anodized GC regions. (f) Variation of Q_c (blue trace) and Γ_{ads} (red trace) with cycle number normalized by the value at the 1st cycle in anodized GC regions.

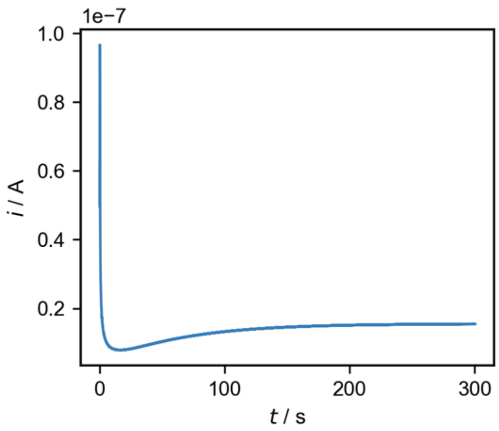

Figure S14. Chronoamperometric (*i*-*t*) curve obtained during the local anodization of the GC surface at +1.5 V (vs Ag/AgCl QRCE) for 300 s using static-mode SECCM. The pipet probe had a ca. 30 µm diameter and was filled with a 5 mM H_2SO_4 solution.

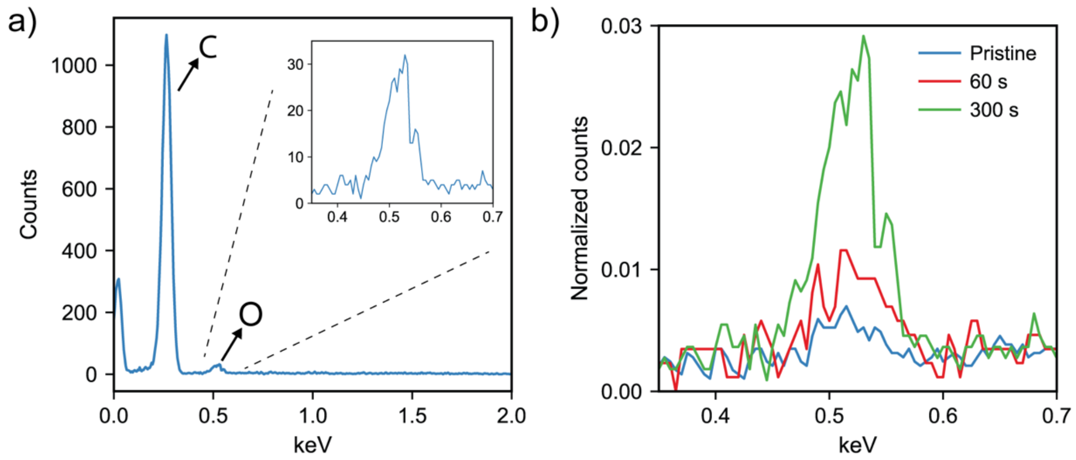

Figure S15. (a) Representative EDS spectrum obtained for GC anodized for 300 s. (b) Oxygen region of EDS spectra for pristine GC (blue trace), anodized for 60 s (red trace) and anodized for 300 s (green trace), normalized by the C signal (i.e., peak maximum for C signal is 1). This representation highlights the O/C ratio between the different surfaces.

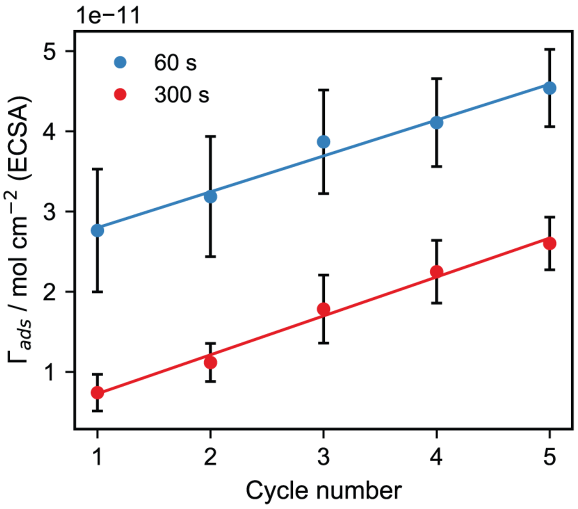

Figure S16. Variation of DA surface concentration (Γ_{ads}) with cycle number in anodized regions of GC after anodization for 60 s (blue trace) and 300 s (red trace). Error bars indicate the standard deviation from all the SECCM pixels for those specific regions. Γ_{ads} values are normalized by the ECSA increment. Solid lines represent fitted linear regression curves with slopes of 4.5±0.4 and 4.9±0.3 pmol cm^{-2} s^{-1} for 60 and 300 s, respectively.

SUPPORTING TABLES

Table S1. Assignment of functional groups in pristine and anodized GC surface by binding energy of the XPS high-resolution C 1s and O 1s spectra.

**High resolution C 1s spectra**							
	**sp^2 C-C**	**sp^3 C-C/C-H**	**C-O-C**	**C=O**	**O=C-O**	**π-π* shake-up**	**C-OH**
**Pristine**	284.41 eV	284.91 eV	286.39 eV	287.8 eV	288.79 eV	290.83 eV	N/A
**Anodized**	284.19 eV	284.89 eV	286.14 eV	287.59 eV	288.78 eV	290.61 eV	286.92 eV

**High resolution O 1s spectra**				
	**O=C**	**O-C**	**Metal oxides**	**Atmos O / H_2O**
**Pristine**	531.94 eV	533.22 eV	530.76 eV	535.07 eV
**Anodized**	531.96 eV	532.86 eV	530.78 eV	535.09 eV

Table S2. Average capacitive charges (Q_c) recorded between -0.375 V and -0.275 V (vs Ag/AgCl QRCE) in pristine (P) and anodized (A) regions of a GC surface, with the ratio between them (A/P) corrected by the stray capacitance. This ratio provides information about the increment of electrochemical surface area after anodization.

**Q_c (average) / fC**	**1st cycle**	**5th cycle**
**Pristine (P)**	30 ± 7	29 ± 7
**Anodized (A)**	82 ± 12	96 ± 13
**Ratio (A/P)**<br>***Corrected by stray capacitance***	3.3	4.1

MOVIE CAPTIONS

Movie S1. Spatially-resolved electrochemical (*i*-*E*) movie (110 pixels over a 55 μm x 50 μm scan area, hopping distance = 5 μm) obtained with the SECCM protocol (1st voltammetric cycle), visualizing the activity of DA oxidation on a GC surface locally anodized for 60 s. The pipet probe contained 30 μM DA in PBS, and scan rate was 47 V s^{-1}.

Movie S2. Spatially-resolved electrochemical (*i*-*E*) movie (110 pixels over a 55 μm x 50 μm scan area, hopping distance = 5 μm) obtained with the SECCM protocol (5th voltammetric cycle), visualizing the activity of DA oxidation on a GC surface locally anodized for 60 s. The pipet probe contained 30 μM DA in PBS, and scan rate was 47 V s^{-1}.
